# Acceptance and commitment therapy for patients with chronic tinnitus resistant to tinnitus retraining therapy: A case series

**DOI:** 10.1002/pcn5.70220

**Published:** 2025-10-12

**Authors:** So Takabatake, Masaki Kondo, Mariko Takahashi, Kayoko Kabaya, Tatsuo Akechi

**Affiliations:** ^1^ Department of Psychiatry and Cognitive‐Behavioral Medicine Nagoya City University Graduate School of Medical Sciences Nagoya Aichi Japan; ^2^ Japan Depression Center Tokyo Japan; ^3^ Department of Otolaryngology, Mirai Kosei Hospital Nagoya City University Graduate School of Medical Sciences Nagoya Aichi Japan; ^4^ Department of Otolaryngology‐Head and Neck Surgery Nagoya City University Graduate School of Medical Sciences Nagoya Aichi Japan

**Keywords:** acceptance and commitment therapy (ACT), cognitive fusion, mindfulness, tinnitus, tinnitus retraining therapy (TRT)

## Abstract

**Background:**

Tinnitus retraining therapy (TRT) promotes habituation in chronic tinnitus but is less effective in patients under psychological distress. Acceptance and commitment therapy (ACT) offers an alternative by enhancing psychological flexibility rather than targeting symptom reduction.

**Case Presentation:**

Five patients with chronic tinnitus unresponsive to TRT completed the ACT program. Among them, four completed the 6‐month follow‐up and are presented in detail. While Case 1 was previously reported, this analysis includes all four to explore ACT's broader application. The intervention targeted ACT's six core processes. Outcomes were measured using the Tinnitus Handicap Inventory (THI), Cognitive Fusion Questionnaire‐7 (CFQ‐7), Valuing Questionnaire (VQ), and Hospital Anxiety and Depression Scale (HADS). After 6 months, three patients showed a clinically meaningful THI reduction. Those without hearing loss improved more in CFQ‐7 and HADS. VQ scores showed minimal change.

**Conclusion:**

ACT may help patients with TRT‐resistant tinnitus, particularly those without hearing loss.

## BACKGROUND

Tinnitus is the perception of sound without an external source and affects 15%–20% of the population, with 2%–3% experiencing distress that disrupts daily life.^1^, ^2^ Severe cases are often associated with anxiety, depression, and insomnia, reducing quality of life.^3^, ^4^

Tinnitus retraining therapy (TRT), which combines sound therapy with directive counseling, is a standard treatment^5^ but is less effective in patients with psychological distress,^6^ highlighting the need for approaches that address tinnitus‐related psychological factors.

Cognitive behavioral therapy (CBT) is effective and recommended in clinical guidelines.^7^, ^8^ Acceptance and commitment therapy (ACT), a CBT‐based approach, promotes acceptance and value‐based actions rather than eliminating tinnitus.^9^, ^10^ Tinnitus‐related distress is closely associated with the perception of tinnitus as a “threat.”^11^ ACT reduces psychological distress by promoting acceptance rather than avoidance.^9^, ^10^ Its efficacy is comparable to CBT,^12^ and it may offer advantages over TRT in reducing tinnitus impact, sleep disturbances, and anxiety.^10^ However, its effectiveness in patients with treatment‐resistant tinnitus unresponsive to TRT remains unclear.

In this study, we applied ACT to five patients with chronic tinnitus unresponsive to TRT and analyzed their clinical course to explore the potential benefits of ACT for TRT‐resistant tinnitus.

## CASE PRESENTATION

### Study design

This retrospective observational study included patients with chronic tinnitus who started ACT at the Neuropsychiatry Department of Nagoya City University Hospital between October 1, 2018, and May 31, 2019. The study was approved by the Institutional Ethics Committee, and all participants provided written informed consent.

### Participants

Adults (20–64 years) with chronic tinnitus ≥ 3 months and persistent distress after ≥6 months of TRT were eligible. Inclusion required a Tinnitus Handicap Inventory (THI) score of ≥18 (indicating mild distress). An otolaryngologist confirmed tinnitus not explained by organic causes. Exclusion criteria were schizophrenia, bipolar disorder, personality disorder, dementia, intellectual disability, and clinically suspected cases of these disorders, as they were considered likely to interfere with the structured ACT program. Major depressive disorder, often comorbid with tinnitus, was not excluded if stable; the same applied to posttraumatic stress disorder. Other exclusion criteria were significant hearing impairment, communication difficulties in Japanese, or concurrent structured psychotherapy. As this was a retrospective case series, the ability to attend weekly 90‐min sessions was not prespecified in the protocol but was explained to patients and served as a practical prerequisite for participation.

All participants were referred from the otolaryngology department of the same institution after tinnitus evaluation. Before enrollment, they had received TRT either at this institution or at external facilities. All participants met the *Diagnostic and Statistical Manual of Mental Disorders, Fifth Edition* (DSM‐5)^13^ criteria for somatic symptom disorder. Five patients completed ACT, but one (Case 5) lacked a 6‐month follow‐up and was excluded from the analysis. Additional details are provided in the Supporting Information, and the characteristics of the four analyzed patients are shown in Table 1.

**Table 1:** Patient characteristics and clinical background.

Case	Age	Sex	Tinnitus	Duration (months)	TRT duration (months)	Etiology	THI baseline (grade)	DSM‐5 Dx	PMH	Medications	ACT sess
1	42	F	Unilateral	40	28	Non‐hearing‐loss tinnitus	24 (Grade 2)	SSD, past MDD	—	Alprazolam 0.4 mg (PRN)	8
2	54	M	Bilateral	93	9	Non‐hearing‐loss tinnitus	44 (Grade 3)	SSD	Enteritis (hosp)	None	7
3	54	M	Bilateral	157	157	Bilateral sensorineural hearing loss (noise‐induced)	92 (Grade 5)	SSD, current MDD	GERD, lacunar infarct (NS)	Ramelteon 8 mg, Eszopiclone 2 mg, Mirtazapine 7.5 mg, Paroxetine 20 mg (QHS)	8
4	57	F	Bilateral	240	168	Suspected otosclerosis (mixed hearing loss)	32 (Grade 2)	SSD, past MDD, past PTSD	Thyroid tumor	Zolpidem 5 mg (PRN)	6

*Note*: Grading according to THI severity: Grade 1 (slight, 0–16), Grade 2 (mild, 18–36), Grade 3 (moderate, 38–56), Grade 4 (severe, 58–76), and Grade 5 (catastrophic, 78–100). Patient demographic and clinical characteristics are summarized in Table 1, including tinnitus etiology, THI baseline severity, psychiatric diagnoses based on DSM‐5, and prescribed medications. This overview provides context for evaluating the psychological flexibility outcomes following ACT.

Abbreviations: ACT, acceptance and commitment therapy; Dx, diagnosis; GERD, gastroesophageal reflux disease; hosp, hospitalized; MDD, major depressive disorder; NS, no sequelae; PMH, past medical history; PRN, as needed; PTSD, posttraumatic stress disorder; QHS, at bedtime; sess, sessions; SSD, somatic symptom disorder; THI, Tinnitus Handicap Inventory; TRT, tinnitus retraining therapy.

### Intervention

**Figure 1:**
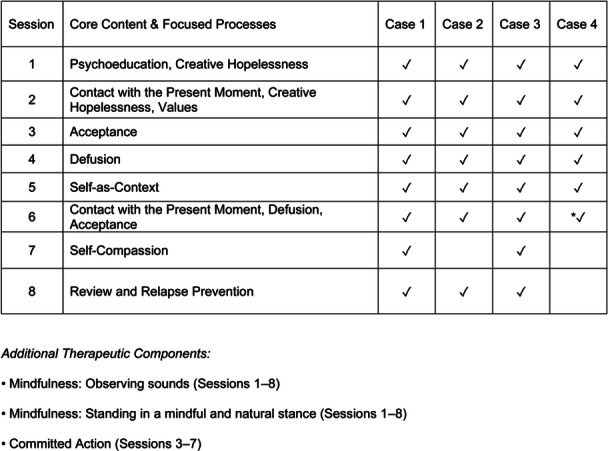
Acceptance and commitment therapy (ACT) session structure and therapeutic components. This figure presents the standard structure of the ACT program across eight sessions. Core psychological processes such as mindfulness, acceptance, and cognitive defusion were progressively introduced. Self‐compassion was included only in selected cases. Each patient received 6–8 weekly 90‐min sessions, with individual modifications. *In Case 4, Session 6 also included review and relapse prevention components. A check mark (✓) indicates that the corresponding component was introduced in the given session. “Defusion” in the figure corresponds to “cognitive defusion” in the text. Additional therapeutic components included martial arts–inspired mindfulness exercises; see Supporting Information for details. *Adapted from* Takabatake and Kondo.^16^ © 2022 Japanese Society of Psychosomatic Medicine.

ACT was delivered by the S.T., a board‐certified psychiatrist with approximately 7 years of clinical experience and approximately 2 years of experience in ACT. The standardized ACT program incorporated six core processes—acceptance, cognitive defusion, contact with the present moment (i.e., being present), self‐as‐context, values, and committed action^14^, ^15^—and integrated Eastern martial arts–based exercises, such as standing in a mindful and natural stance and observing sounds with a “diffuse gaze” adapted from the martial arts concept of *metsuke* (broad, unfocused gaze), as previously described^16^ (see Figure 1 and Supporting Informaton). Details of the specific ACT core processes applied to each case in each session are presented in Figure 1. The program consisted of eight weekly 90‐min sessions, with individual modifications. Self‐compassion‐based interventions^17^ were introduced in Cases 1 and 3; however, in the early cases, this component tended to evoke emotional discomfort related to past experiences unrelated to tinnitus. To prevent reinforcing such non‐tinnitus‐related distress and to maintain therapeutic focus on tinnitus‐related processes, this component was omitted in subsequent cases (Cases 2, 4, and 5). As a result, Case 2 received seven sessions, whereas Cases 4 and 5 each received six sessions due to individual circumstances. Although the self‐compassion and review sessions were omitted, the overall program structure and texts remained consistent. In both Cases 4 and 5, Session 6 also incorporated review and relapse prevention components, ensuring continuity despite the reduced number of sessions. The two foundational mindfulness exercises were retained, with slightly increased emphasis on process‐oriented experiential practices at the beginning of each session.

**Figure 2:**
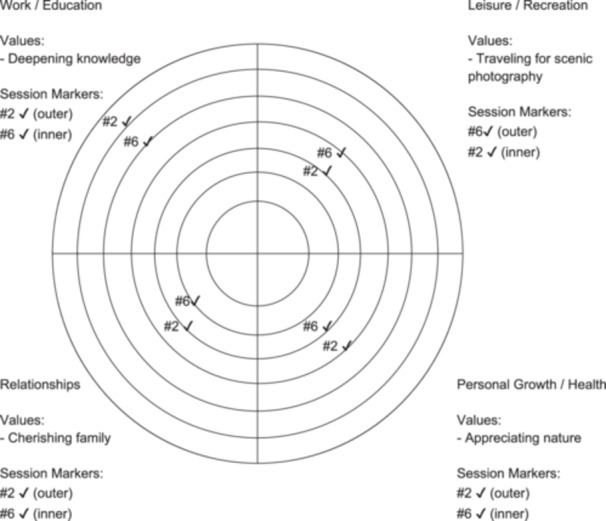
Bull's Eye exercise—Case 2. This figure illustrates value alignment across four life domains (work/education, leisure, relationships, and personal growth) using the structured Bull's Eye exercise. Personalized value statements are presented for each domain. Session markers from Session 2 and Session 6 indicate changes in perceived alignment with these values over time. Proximity to the center represents stronger alignment with personal values. ✓ Closer to the center = greater alignment with personal values.

Sessions emphasized the paradoxical nature of tinnitus control strategies and facilitated mindfulness‐based, nonevaluative sound observation.^22^, ^23^, ^24^, ^25^ Value‐based behavioral activation was reinforced through exercises, with the Bull's Eye exercise^26^ assigned as homework to align personal values with daily behavior (Figure 2).

### Outcome measures

The primary endpoint was tinnitus distress at 6 months post‐intervention, assessed using the THI (range: 0–100), categorized from Grade 1 (slight) to Grade 5 (catastrophic).^27^, ^28^, ^29^ Secondary outcomes included cognitive fusion, assessed using the Cognitive Fusion Questionnaire‐7 (CFQ‐7)^18^, ^19^; value‐based actions, assessed using the Valuing Questionnaire (VQ),^20^, ^21^ with VQ‐Progress measuring value‐consistent behavior and VQ‐Obstruction assessing interference from internal barriers; and anxiety and depression, assessed using the Hospital Anxiety and Depression Scale (HADS).^30^, ^31^ All outcomes were evaluated at baseline and at 1, 3, and 6 months post‐intervention, and all instruments were validated Japanese versions. Outcome significance was determined using the minimal clinically important difference (MCID) for THI (7 points^32^) and HADS (approximately 1.5 points^33^), and the reliable change index (RCI > 1.96) for CFQ‐7 and VQ.^34^ For the Japanese CFQ‐7, which lacks test–retest data, Cronbach's *α* was used as a surrogate.

### Case summaries

Detailed case descriptions are provided for Cases 2 and 3, as they are representative of different patterns of treatment response. Case 1 was previously reported,^16^ and the remaining cases are summarized in the Supporting Information.

#### Case 2 (54‐year‐old man with tinnitus without hearing loss)

At age 52, after work‐related stress, a man developed persistent tinnitus that had previously been intermittent. Otolaryngology confirmed normal hearing; magnetic resonance imaging (MRI) and computed tomography (CT) were unremarkable. Stress and tinnitus led him to retire from work. He received TRT for 9 months, but distress, anxiety, and avoidance of valued activities (e.g., travel) persisted. At 54, he began ACT and completed seven sessions. He initially struggled with intrusive thoughts and attempts to control tinnitus, but through mindfulness learned to observe breath and sounds, including tinnitus and related thoughts, without alteration. His core values included “deepening knowledge,” “cherishing family,” “appreciating nature,” and “traveling for scenic photography” (Figure 2). Over time, distress decreased and value‐based actions increased. At 1 month, tinnitus remained but anxiety had declined; at 3 months, he reported greater psychological flexibility; by 6 months, he no longer experienced tinnitus‐induced anxiety, describing it as “just something that exists.” He resumed meaningful activities, including cycling, advising his son, and traveling with his wife.

Outcome: The THI score improved from 44 (Grade 3) to 28 (Grade 2), and CFQ‐7 score decreased, indicating reduced cognitive fusion.

#### Case 3 (54‐year‐old man with tinnitus and sensorineural hearing loss)

Around age 40, the patient developed bilateral tinnitus during workplace stress. At 53, he developed sensorineural hearing loss from prolonged occupational noise exposure (grinding work), which exacerbated tinnitus. He later moved to a sales position but resigned after witnessing workplace harassment, which heightened psychological distress. Despite over a year of TRT with hearing aids and sound generators, along with 10 months of acupuncture and pharmacological treatment with antidepressants and anxiolytics, improvement was minimal. He then opted for ACT and completed eight sessions. Mindfulness exercises facilitated a more neutral observation of tinnitus, reducing preoccupation and its impact. He identified “enjoying time with others while smiling together” as a core value, which motivated renewed social engagement. He also pursued new challenges, including resume preparation and driving, despite anxiety.

Outcome: At 1 month, the THI score improved from 92 (Grade 5) to 56 (Grade 3), with reduced preoccupation. However, assuming a homemaker role against his wishes worsened tinnitus, and the THI score returned to 84 (Grade 5) at 6 months.

Six months posttreatment, four of the five patients completed follow‐up (Table 2). Three cases (1, 2, and 3) met the MCID threshold for THI (≥7‐point reduction), indicating improved tinnitus distress. The two cases without hearing loss (Cases 1 and 2) also showed improved THI severity and met the MCID for HADS‐Depression, with one also meeting the MCID for HADS‐Anxiety. Case 4 (mixed hearing loss) showed no sustained improvement in THI, although the severity grade remained stable at Grade 2. CFQ‐7 RCI exceeded 1.96 in Cases 1 (RCI = −2.69) and 2 (RCI = −3.50), indicating significant cognitive fusion reduction. While VQ‐Progress and VQ‐Obstruction did not meet RCI criteria in any case, the Bull's Eye exercise used as a homework assignment showed movement toward the center during the session period in each case (Table 3).

**Table 2:** Longitudinal changes in tinnitus distress and psychological measures in four cases.

Measure	Time point	Case 1	Case 2	Case 3	Case 4
THI	Baseline	24	44	92	32
	Post	22	30	58	22
	1M	26	40	56	18
	3M	14	28	76	20
	6M	6	28	84	36
CFQ‐7	Baseline	39	27	28	14
	Post	30	25	24	13
	1M	35	23	26	13
	3M	29	25	26	13
	6M	29	14	26	21
VQ‐P	Baseline	8	12	13	12
	Post	12	16	13	15
	1M	12	16	12	21
	3M	15	15	12	15
	6M	14	16	11	15
VQ‐O	Baseline	20	10	16	6
	Post	16	11	13	5
	1M	19	14	15	11
	3M	20	12	16	4
	6M	17	6	16	9
HADS‐A	Baseline	9	9	12	0
	Post	8	6	7	2
	1M	8	9	11	1
	3M	8	11	10	4
	6M	4	11	10	8
HADS‐D	Baseline	10	10	17	1
	Post	5	6	10	1
	1M	5	8	9	3
	3M	4	6	12	2
	6M	5	5	10	5

*Note*: Scores are presented at five time points: baseline, post‐intervention, and at 1‐, 3‐, and 6‐month follow‐up. Reliable change was defined as RCI > 1.96, based on psychometric properties of CFQ‐7 (SD = 8.76, Cronbach's *α* = 0.91, mean = 27.57),^18^, ^19^ VQ‐P (SD = 5.32, ICC = 0.824, mean = 15.9) and VQ‐O (SD = 4.55, ICC = 0.641, mean = 16.6).^20^, ^21^ Because Japanese CFQ lacks test–retest reliability, Cronbach's *α* was used for consistency.

Abbreviations: CFQ‐7, Cognitive Fusion Questionnaire‐7 (range 0–49, higher scores indicate greater cognitive fusion); HADS, Hospital Anxiety and Depression Scale, including HADS‐A (Anxiety, range 0–21) and HADS‐D (Depression, range 0–21), with higher scores indicating greater symptom severity; THI, Tinnitus Handicap Inventory (range 0–100, higher scores indicate greater tinnitus distress); VQ, Valuing Questionnaire, including VQ‐P (Progress, range 0–30, higher scores indicate greater alignment with values) and VQ‐O (Obstruction, range 0–30, higher scores indicate greater obstruction to valued actions).

**Table 3:** Bull's Eye score progression across sessions.

Case number	Session 2	Session 5	Session 6	Session 7
Case 1	5.5	‐	‐	10.5
Case 2	16.0	‐	18.0	‐
Case 3	7.0	‐	‐	12.0
Case 4	18.0	20.0	‐	‐

*Note*: Bull's Eye scores indicate proximity to the center, representing value‐aligned actions. Scores range from 1 (outermost ring) to 7 (center), reflecting increasing alignment with personal values. The overall score represents the sum of the four domain scores (range 4–28), and an improvement of three points or more in this total score is considered clinically significant.^26^ Scores are calculated based on the distance from the center of the target, with higher scores indicating greater proximity to the center. If the mark is positioned between two score zones, the score is calculated as the midpoint value (e.g., between 3 and 4, the score will be 3.5). To maintain consistency, all scores are presented to one decimal place.

## DISCUSSION

This case series describes the application of ACT in five patients with chronic tinnitus resistant to TRT. All participants completed the ACT program, demonstrating its feasibility. However, only four patients completed the 6‐month follow‐up and were included in the outcome analysis. In cases without hearing loss (Cases 1 and 2), improvements in both the THI and the CFQ‐7 were observed, indicating a reduction in psychological distress. In Case 2, the HADS‐A score slightly increased, but the THI improved, suggesting that general anxiety scales may miss tinnitus‐specific changes, underscoring the value of condition‐specific measures like the THI. In contrast, among cases with hearing loss (Cases 3 and 4), although some showed improvements in THI scores, none met the RCI criteria for CFQ‐7, suggesting limited behavioral change following ACT. ACT efficacy appears influenced by tinnitus‐related cognitive‐emotional factors.

Our findings align with prior research supporting ACT efficacy in tinnitus without hearing loss. Westin et al.^10^ reported that ACT was more effective than TRT in individuals with tinnitus without hearing impairment. Similarly, in this study, improvements in THI and CFQ‐7 scores were observed in cases without hearing loss, suggesting that a reduction in cognitive fusion regarding tinnitus contributed to alleviating psychological distress. Tinnitus distress is predominantly cognitive‐emotional.^7^, ^11^ Patients with tinnitus without hearing loss may be more responsive to ACT's focus on changing their relationship with thoughts, as they cannot directly associate the tinnitus sound with hearing impairment.

Conversely, the effectiveness of ACT in patients with tinnitus accompanied by hearing loss was limited. Jastreboff and Jastreboff^35^ noted that when external sounds are reduced, the contrast between tinnitus‐related neural activity and background activity increases, making the perception of tinnitus more prominent. In this study, patients with hearing loss were more preoccupied with their tinnitus, and improvements in THI scores were limited, suggesting that ACT alone may not be sufficient. Instead, a combination of ACT with sound therapy or auditory rehabilitation may be necessary.

Tinnitus distress is often amplified by negative cognitive appraisals of tinnitus.^11^ Therefore, a key component of ACT is to reduce excessive cognitive engagement with tinnitus by fostering nonjudgmental awareness through defusion.^36^ In this study, patients were instructed to “gaze blankly” at sounds rather than actively “listen,” as judging sounds as unpleasant can hinder mindfulness. This approach facilitated labeling the perception simply as a “sound.” In cases without hearing loss, it may have contributed to reducing cognitive fusion and improving CFQ‐7 scores.

Limitations include the small sample size, lack of follow‐up data for Case 5, and the use of the Japanese version of the VQ—validated in Japanese university students—which may not fully capture value‐based changes in chronic tinnitus patients, highlighting the need for more suitable clinical measures.

## CONCLUSION

ACT may be effective for TRT‐resistant tinnitus. Further research with larger samples is needed.

## AUTHOR CONTRIBUTIONS

All authors contributed to the conception and design of the study. So Takabatake conducted the ACT sessions, collected data, performed statistical analyses, and drafted the original manuscript. Mariko Takahashi and Kayoko Kabaya contributed to patient recruitment and clinical assessments. So Takabatake, Masaki Kondo, Mariko Takahashi, Kayoko Kabaya, and Tatsuo Akechi critically reviewed and revised the manuscript. All authors approved the final version of the manuscript.

## CONFLICT OF INTEREST STATEMENT

Tatsuo Akechi, Vice Editor‐in‐Chief of *Psychiatry and Clinical Neurosciences Reports* and coauthor of this article, was not involved in the editorial decision regarding its acceptance and publication. Drs. So Takabatake, Masaki Kondo, and Mariko Takahashi have received funding from Olive Union Corporation. The remaining authors declare no conflicts of interest.

## ETHICS APPROVAL STATEMENT

This study was approved by the Institutional Review Board of Nagoya City University Hospital (Approval No. 60‐23‐0029). All procedures were conducted in accordance with the ethical standards of the Declaration of Helsinki.

## PATIENT CONSENT STATEMENT

Written informed consent was obtained from all participants for publication of this case series.

## CLINICAL TRIAL REGISTRATION

N/A.

## Supporting information

Supporting Information.

## Data Availability

The data for this study are not publicly available due to privacy and ethical restrictions.
