# Supplementary material for: Acceptance and commitment therapy for patients with chronic tinnitus resistant to tinnitus retraining therapy: A case series
Source: PCN Rep. 2025 Oct 12;4(4):e70220. doi: 10.1002/pcn5.70220 (PMC12515982; doi:10.1002/pcn5.70220)
Supplement: Supplementary file 1 — Supporting Information. [file PCN5-4-e70220-s001.pdf]

Supplementary Material: Case Descriptions and Mindfulness Exercises

This document provides detailed case descriptions for Cases 1 and 4 (omitted from the main text due to space constraints) and Case 5 (excluded from the outcome analysis because 6-month follow-up data were unavailable). It also includes descriptions of martial arts–inspired mindfulness exercises that were part of the ACT program.

Case 1 (42-year-old woman with tinnitus without hearing loss)
At the age of 39 years, the patient developed left-sided tinnitus and hyperacusis while undergoing infertility treatment. Although her Tinnitus Handicap Inventory (THI) score temporarily improved with TRT, she continued to experience hypersensitivity to everyday sounds and persistent anxiety about worsening tinnitus, leading her to take a leave of absence from her part-time job. She became highly engaged in yoga to control her tinnitus, but this approach paradoxically intensified her distress. Seeking an alternative approach that did not focus on controlling tinnitus, she opted for ACT and participated in eight sessions.
During ACT, the patient identified and clarified her core values across four domains: work, relationships, personal growth, and leisure. Her key values included "living mindfully," "spending time with people with whom she can be her authentic self," "appreciating everyday happiness," and "connecting with nature and enjoying the seasons." The intervention encouraged her to engage in behaviors aligned with these values. As the sessions progressed, her engagement in value-driven actions increased. At the 6-month follow-up, she reported that "acting in accordance with my values has become natural." She resumed social activities with friends and returned to her part-time job. Additionally, her hyperacusis symptoms improved, allowing her to visit noisy locations without discomfort.
Outcome: The THI score decreased from 24 (Grade 2) to 6 (Grade 1), and the CFQ-7 score improved.

Case 4 (57-year-old woman with bilateral tinnitus and mixed hearing loss)
The patient developed tinnitus at the age of 38 years due to social stress. She received TRT at 44 years old, achieving partial improvement but continued to experience fluctuations related to psychological stress. She reported chronic stress from family issues and physical symptoms, such as gastric discomfort. She relied on zolpidem (5 mg) for sleep and reported distress over her inability to sleep without medication.
She underwent six ACT sessions, practicing mindfulness-based sound observation and

recognizing that various sounds simply exist without needing evaluation. She also identified key values: "cherishing family and friends" and "enjoying golf and music."
At 1-month follow-up, she recognized she was overly fixated on sleep and began engaging in value-based activities, like golf. She reduced her zolpidem intake to 2.5 mg, with some medication-free nights. However, external factors later worsened her condition.
Outcome: THI remained stable at Grade 2 without clinically significant change.

Case 5 (54-year-old man with tinnitus due to sudden sensorineural hearing loss)
The patient developed right-sided tinnitus following sudden sensorineural hearing loss (SSNHL) at 50 years of age. Steroid treatment was ineffective. He experienced fluctuating hearing loss with autophony, and TRT with hearing aids was unsuccessful (THI = 92). He also avoided speaking due to the "unpleasant resonance of his own voice" and developed panic-like symptoms in trains and long drives. Despite trials of antidepressants and other treatments, tinnitus did not improve. His psychiatric diagnoses included somatic symptom disorder, major depressive disorder (current episode), and agoraphobia. At the time of ACT, he was taking mirtazapine 30 mg, eszopiclone 2 mg at bedtime, sulpiride 100 mg twice daily, and clotiazepam 5 mg as needed.
He underwent six ACT sessions, but his focus remained on oral discomfort rather than tinnitus. Although he acknowledged that "accepting discomfort requires relaxation rather than tension," he struggled to sustain this approach after therapy.
Outcome: At 3 months, the THI score improved from 84 (Grade 5) to 62 (Grade 4), but the CFQ-7 score worsened. Because 6-month follow-up data were unavailable, this case was excluded from the analysis and is presented only in the Supplementary Material.

Description of martial arts–inspired mindfulness exercises
This ACT program included mindfulness exercises inspired by traditional Eastern martial arts. Two core practices were conducted in all sessions:

1. Standing in a mindful and natural stance
Participants stood with feet shoulder-width apart, head gently suspended from above, and attended to bodily sensations of relaxation and a settled center of gravity below the navel. This posture reflects the “natural stance” in traditional martial arts and served as the starting point for embodied awareness.

2. Observing sounds

Building on this embodied awareness, participants then extended their attention outward to auditory experiences. They were instructed to adopt a “diffuse gaze” toward ambient sounds while seated or standing, as if observing sounds floating in the surrounding space rather than actively listening. This exercise was inspired by the martial arts concept of *metsuke* (broad, unfocused gaze). In particular, it is comparable to *tooyama no metsuke* (also pronounced *enzan no metsuke*, literally “gazing at a distant mountain”), which avoids fixation on any single point by maintaining a wide visual field. By analogy, participants were guided to experience tinnitus and other sounds in a nonjudgmental manner, thereby supporting ACT processes such as defusion and acceptance.

Other martial arts–based experiential exercises were included in the program; the full intervention protocol is described in detail in Takabatake & Kondo (2022, in Japanese).
